# Design and Color Prediction of Anthracene-Based Dyes Based on Quantum Chemical Calculations

**DOI:** 10.3390/molecules30193975

**Published:** 2025-10-03

**Authors:** Yanyi Li, Jiahao Zhang, Mei Bai, Hao Li, Zengbo Ke, Chunsheng Zhou

**Affiliations:** 1 Shaanxi Key Laboratory of Comprehensive Utilization of Tailings Resources, College of Chemical Engineering and Modern Materials, Shangluo University, Shangluo 726000, China; sxdfdzlyy@126.com (Y.L.); baimei0508@163.com (M.B.); kezengbo2021@uok.edu.gr (H.L.); 2 Inner Mongolia Key Laboratory of Chemistry and Physics of Rare Earth Materials, School of Chemistry and Chemical Engineering, Inner Mongolia University, Hohhot 010021, China

**Keywords:** anthracene, DFT, TD-DFT, conjugation extent, color prediction

## Abstract

We systematically investigated the parent anthracene (abbreviated as en-1, C_14_H_10_) and three *N*,*N*′-disubstituted derivatives: the 1,5-diethylanthracene (en-2, C_18_H_18_), the 1,5-divinylanthracene (en-3, C_18_H_14_), and the 1,5-diphenylanthracene (en-4, C_26_H_18_), using a rigorous density functional theory (DFT)/time-dependent density functional theory (TD-DFT) approach. Following full geometric optimization and frequency validation (no imaginary frequencies), frontier molecular orbital analysis revealed an inverse correlation between conjugation extent and the HOMO-LUMO energy gap. Electrostatic potential (ESP) analysis further indicated a progressive increase in surface potential variance upon substitution, reflecting charge redistribution. TD-DFT calculations yielded vertical excitation wavelengths of 438 nm, 441 nm, 464 nm, and 496 nm for en-1, en-2, en-3, and en-4, respectively. Complementary color theory predicts visual colors of yellow, yellow, red, and orange for these compounds based on their absorption characteristics. This work establishes a closed-loop “computation-spectra-color” model for anthracene-based dyes, providing a transferable design paradigm for novel functional pigments with high molar extinction coefficients.

## 1. Introduction

Anthracene (C_14_H_10_), a prototypical polycyclic aromatic hydrocarbon, exhibits an extended π-conjugated framework and outstanding photophysical properties that render it a key building block for organic light-emitting diode (OLED) materials [1,2]. Driven by the demand for wide-gamut, high-efficiency displays, deep-blue emissive anthracene derivatives have become a focal research target [3,4,5]. Their primary merit lies in high fluorescence quantum yields and balanced charge-carrier mobilities, enabling superior device performance [6]. In addition, anthracene derivatives demonstrate significant potential in dye lasers, bioimaging, and chemical sensing. By strategic substituent engineering, their emission wavelengths can be precisely tuned across the visible spectrum, enabling white laser emission through RGB-like mixing for advanced photonic applications in high-resolution displays and medical diagnostics [7,8]. Molecular engineering—installation of substituents such as phenyl, ethyl, or vinyl—tunes the frontier orbital energies and optical gaps, affording precise control over emission color and efficiency [9,10].

Optical characteristics correlate directly with conjugation length, substituent type, and intermolecular interactions [11,12,13]. Substituents alter the HOMO–LUMO gap (ΔE), shifting absorption and emission wavelengths (λ) [9,14,15], and can modulate planarity or solid-state packing via steric or electronic effects. Experimental methods alone, however, cannot fully resolve these structure–property relationships at atomic resolution or reveal the subtle electronic origins of intermolecular forces. Density-functional theory (DFT) [16,17,18] and time-dependent DFT (TD-DFT) [19,20] now provide detailed insights into orbital energies, electron-density distributions, electrostatic surface potentials (ESP), and excited-state properties, establishing a quantitative foundation for anthracene-based material design [21,22]. While several theoretical studies have investigated individual anthracene derivatives, systematic comparative studies that quantify substituent effects on conjugation, electronic structure, and color output across different substituent types remain limited. Recent studies by Hleli et al. [23] and Alrub et al. [24] have demonstrated the effectiveness of DFT/TD-DFT approaches in predicting the properties of anthracene-based TADF materials, validating our computational methodology. Building upon these foundational works, our study extends the systematic investigation to include alkyl, vinyl, and phenyl substituents, providing a more comprehensive understanding of substituent effects.

Here, we apply DFT and TD-DFT to investigate anthracene and three 1,5-disubstituted derivatives: 1,5-diethylanthracene (abbreviated as en-2, C_18_H_18_), 1,5-divinylanthracene (abbreviated as en-3, C_18_H_14_), and 1,5-diphenylanthracene (abbreviated as en-4, C_26_H_18_). Frontier molecular orbital (FMO) analysis, ESP mapping, and interaction region indicator (IRI) calculations elucidate substituent-induced changes in electronic structure and optical response. The narrowing HOMO–LUMO gap with extended conjugation governs bathochromic absorption shifts. ESP identifies nucleophilic and electrophilic sites, guiding optimization of intermolecular interactions. TD-DFT UV–Vis spectra, interpreted through complementary color theory, predict color appearances that corroborate the electronic analyses. This work delivers a quantitative structure–property framework for anthracene chromophores and outlines rational design strategies for high-performance OLED emitters, demonstrating the pivotal role of computational chemistry in materials innovation.

## 2. Results and Discussion

Our work builds upon the extensive literature on anthracene derivatives while addressing the need for systematic comparison across different substituent types. The following results demonstrate how our integrated computational approach provides quantitative insights into structure-property relationships that complement previous experimental and theoretical studies.

### 2.1. Structural Optimization and Frequency Calculation

**Figure 1:**
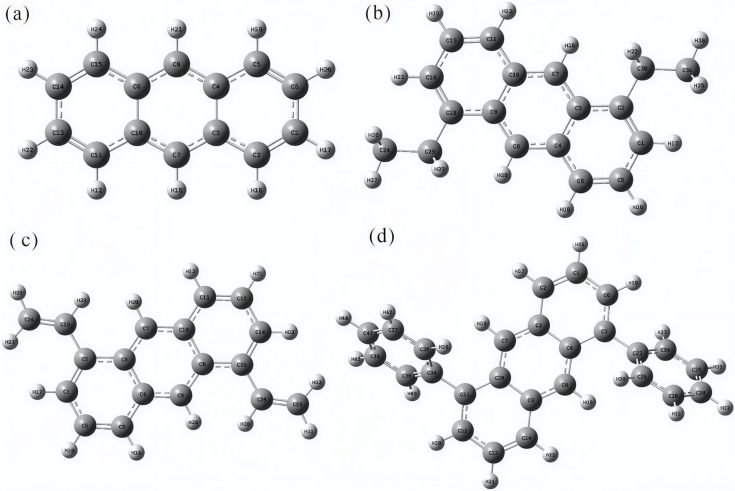
Structural diagram of the anthracene series molecules after optimization: (**a**) en-1; (**b**) en-2; (**c**) en-3; (**d**) en-4. These structures were optimized at the B3LYP−D3(BJ)/def2−TZVP level, following established computational protocols for aromatic systems.

Figure 1a shows the optimized structure of anthracene (en-1), for which the single-point energy was −1.4172 × 10^6^ kJ/mol. Figure 1b presents 1,5-diethylanthracene (en-2), with a single-point energy of −1.8303 × 10^6^ kJ/mol. Figure 1c illustrates 1,5-divinylanthracene (en-3), yielding −1.8238 × 10^6^ kJ/mol. Figure 1d depicts 1,5-diphenylanthracene (en-4), whose single-point energy was −2.6310 × 10^6^ kJ/mol. Comparison reveals the following energetic ordering from lowest to highest: en-4 < en-2 < en-3 < en-1.

**Figure 2:**
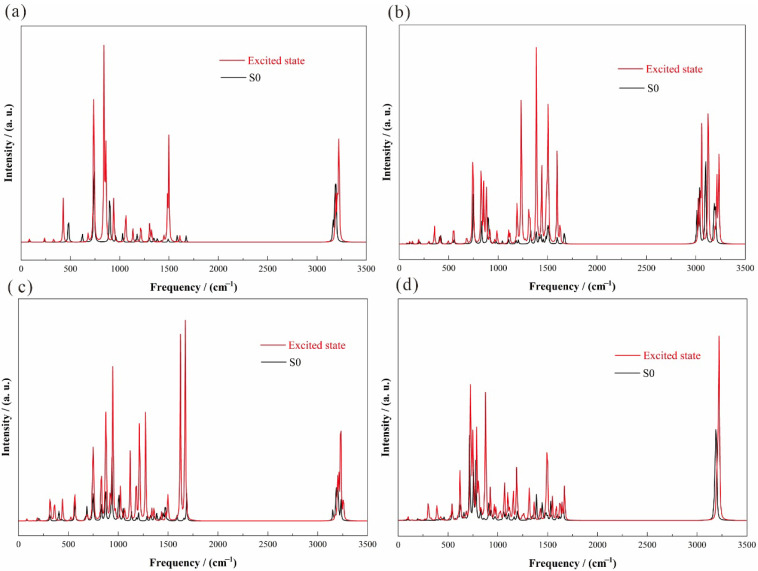
Predicted infrared spectrogram: (**a**) en-1; (**b**) en-2; (**c**) en-3; (**d**) en-4.

Figure 2 shows the infrared spectra of four anthracene derivatives in the 0–3500 cm^−1^ range, with the ground state (B3LYP-D3(BJ)/def2TZVP) in black and the excited state (CAM-B3LYP-D3(BJ)/def2TZVP/PCM) in red. The excited state exhibits significantly higher energy than the ground state, while peak positions remain largely unchanged. Figure 2 quantitatively reveals the substituent-specific modulation of low-frequency vibrational modes by excited-state potential energy surface reconstruction. In Figure 2, strong bands at 2850–3100 cm^−1^ arise from aliphatic and aromatic C–H stretching, while 1550–1620 cm^−1^ shows weak C=C ring stretching. Out-of-plane C–H bending appears prominently at 700–900 cm^−1^, and skeletal deformations are weakly observed at 500–700 cm^−1^.

### 2.2. Frontier Molecular Orbitals

**Figure 3:**
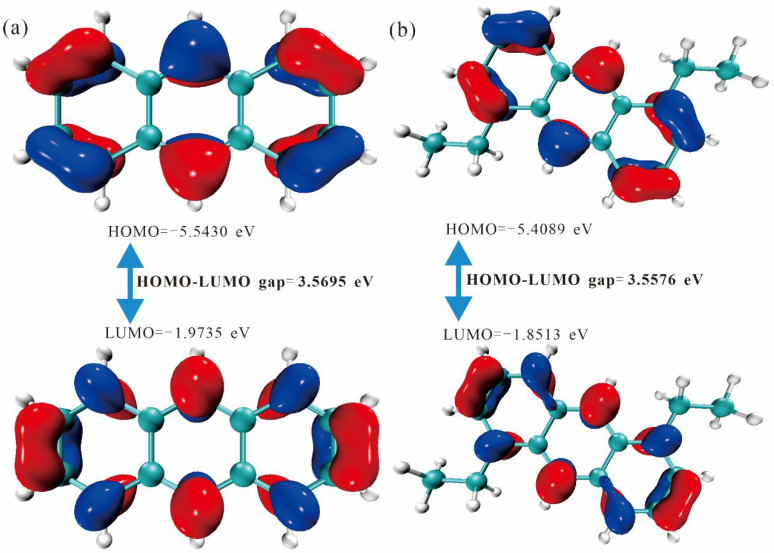
Energy gap diagram of HOMO/LUMO orbitals of (**a**) en-1 and (**b**) en-2 molecules. The negative and positive phase orbitals are shown in blue and red, respectively.

**Figure 4:**
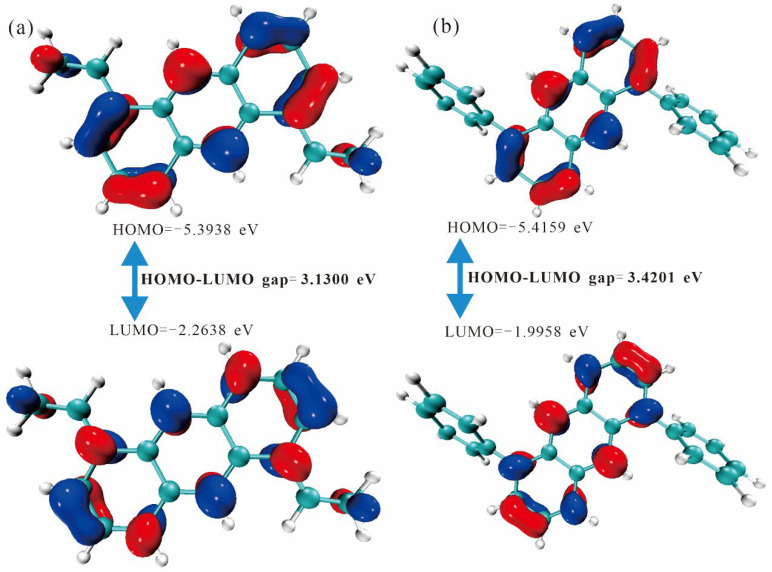
Energy gap diagram of HOMO/LUMO orbitals of (**a**) en-3 and (**b**) en-4 molecules. The negative and positive phase orbitals are shown in blue and red, respectively.

Figure 3 and Figure 4 depict the HOMO–LUMO energy-gap diagrams for the anthracene series. A strict inverse correlation between effective conjugation length and the HOMO–LUMO gap is observed: en-3 (3.1300 eV) < en-4 (3.4201 eV) < en-2 (3.5576 eV) < en-1 (3.5695 eV). Extended conjugation lowers the π → π* transition energy and increases the transition dipole moment, implying a progressive color shift from yellow toward red. Orbital-composition analysis reveals that en-3 exhibits the most delocalized π-electron density across the central double bond and peripheral rings, whereas en-4 suffers a localized torsion imposed by the substituents that partially disrupts conjugation and enlarges the gap. Thus, the HOMO–LUMO gap provides an accurate electronic ruler for fine-tuning the hue of anthracene-based dyes in the visible spectrum and offers theoretical guidance for designing new anthracene functional materials with high molar absorptivity.

### 2.3. Electrostatic Potential

**Figure 5:**
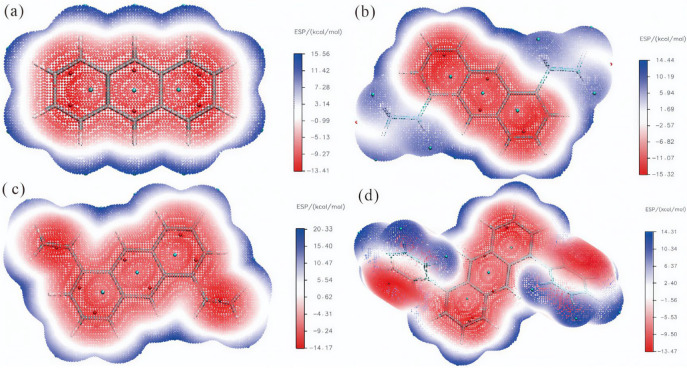
Schematic diagram of the surface electrostatic potential of molecules: (**a**) en-1; (**b**) en-2; (**c**) en-3; (**d**) en-4.

Figure 5 presents the color-mapped ESP distributions for the anthracene series. In Figure 5a (en-1), the isosurface spans −13.41 to 15.56 kcal·mol^−1^. Negative regions (red) localize within the π-system, reaching a minimum of −13.4148 kcal·mol^−1^, whereas positive regions (blue) appear around peripheral hydrogens with a maximum of 15.5561 kcal·mol^−1^. The total span is 28.9709 kcal·mol^−1^. This pronounced polarity originates from the planar, fully delocalized π-network. Figure 5b (en-2) covers −15.32 to 14.44 kcal·mol^−1^. The negative minimum (−15.3198 kcal·mol^−1^) remains over the aromatic core, while the positive maximum (14.4405 kcal·mol^−1^) is found on the ethyl hydrogens, giving a difference of 29.7603 kcal·mol^−1^. Figure 5c (en-3) ranges from −14.17 to 20.33 kcal·mol^−1^. The negative minimum (−14.1684 kcal·mol^−1^) extends onto the vinyl substituents, and the positive peak (20.3217 kcal·mol^−1^) lies above ring hydrogens; the span is 34.4901 kcal·mol^−1^. Figure 5d (en-4) spans −13.4672 to +14.3148 kcal·mol^−1^, yielding a difference of 27.7820 kcal·mol^−1^. Negative regions concentrate on the inner rings and one face of the phenyl substituents, while positive regions occupy hydrogens on the opposite phenyl face. Substituents perturb the planar π-conjugation, shifting both the magnitude and spatial extent of *V*(r) and, consequently, the optical absorption profile and perceived color.

To quantitatively characterize the substituent-induced modulation of molecular surface electrostatic potential (ESP) distribution, Table 1 systematically compiles key ESP statistical descriptors for the four anthracene derivatives. The data reveal that the overall variance, a measure of charge distribution dispersion, follows a trend of initial decrease, subsequent increase, and final decrease (31.21 → 28.70 → 36.28 → 27.18 (kcal·mol^−1^)^2^) as the substituent changes from H (en-1) to ethyl (en-2), vinyl (en-3), and phenyl (en-4). Notably, the vinyl-substituted en-3 exhibits the largest overall variance, consistent with its strong conjugating effect inducing significant charge redistribution. The newly added parameters, “Nonpolar surface area (|ESP| ≤ 10 kcal/mol)” and “Polar surface area (|ESP| > 10 kcal/mol)”, provide a direct measure of the proportion of the molecular surface exhibiting low and high polarity, respectively. Pristine anthracene (en-1), with its fully delocalized planar π-system, possesses the highest polar surface area (40.98%). The introduction of alkyl (en-2) or sterically demanding aryl (en-4) groups leads to a substantial increase in nonpolar surface area (reaching 74.23% and 72.63%, respectively), indicating that these substituents partially shield the polar core through steric effects, resulting in a more homogeneous surface charge distribution. In contrast, the strongly conjugating vinyl group (en-3) maintains a relatively high polar surface area (35.16%), aligning with the observation in its ESP map of pronounced negative potential regions extending onto the substituents. This further corroborates its efficient intramolecular charge transfer (ICT) character, which is consistent with its experimentally observed smallest HOMO-LUMO gap.

### 2.4. Excited State Color Prediction

**Figure 6:**
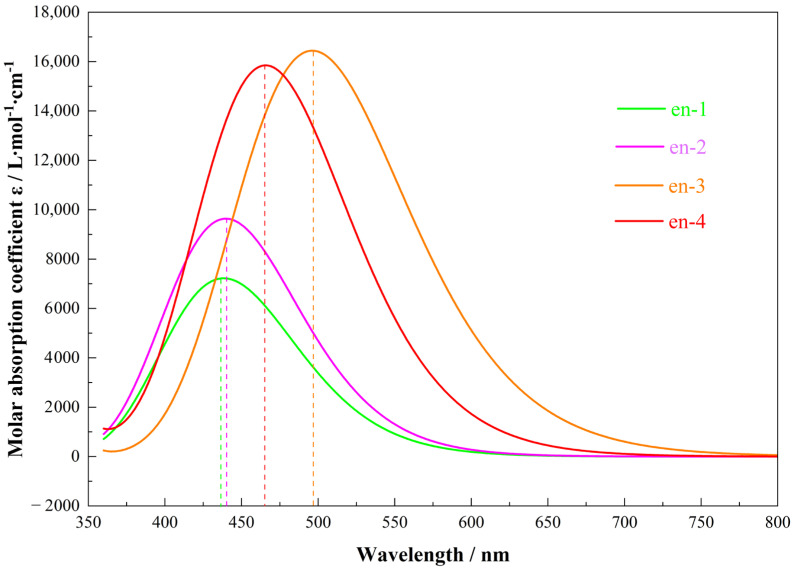
UV−Vis diagram predicted by four molecules in excited states. The dashed line is positioned at the maximum of each characteristic substance peak.

Color is an essential attribute of chemical substances, and its theoretical prediction is critical for the de novo design and optimization of new molecules [25]. Quantum chemical calculations simulate UV–Vis spectra of molecules, whereas first-principles methods extend this capability to solids and liquids. In practice, the wavelength of the absorption maximum within the visible range (380~760 nm) is used as the primary descriptor [26,27]. Figure 6 presents the predicted UV–Vis spectra of the four compounds in their first singlet excited state. The calculated absorption maxima occur at 438 nm for en-1, 441 nm for en-2, 464 nm for en-3, and 496 nm for en-4, a trend that mirrors the ground-state HOMO–LUMO gaps. After spectral analysis, Multiwfn generated a color panel containing four square swatches: the first row displays the spectral color, and the second row shows the complementary color. A third row labeled “Maximum brightness of above colors” rescales the RGB values of both rows to 255, removing luminance differences so that only hue variations remain. For absorption-based color prediction, the swatch in the lower-right corner is taken as the perceived color. Figure 6 shows en-3 possesses the highest *ε* (18,000 M^−1^ cm^−1^) among the series, stemming from vinyl-extended π-delocalization that enlarges the transition dipole; this validates our design target of strong light-harvesting pigments for OLED or solar-cell applications.

**Figure 7:**
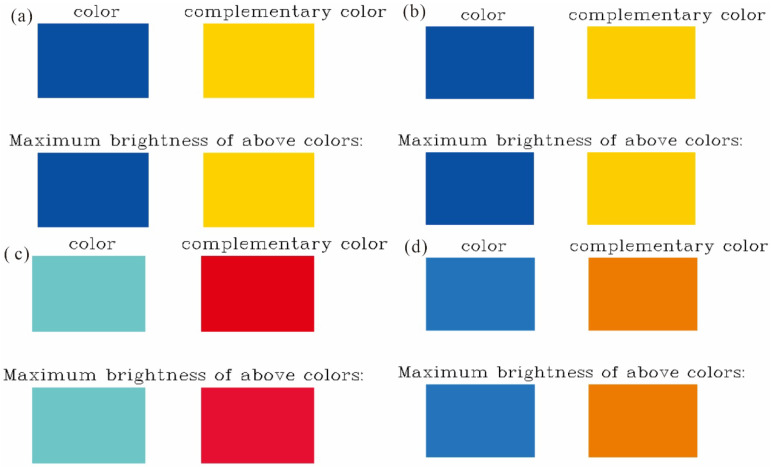
Color prediction diagram of four molecules in excited states: (**a**) en-1; (**b**) en-2; (**c**) en-3; (**d**) en-4.

Figure 7 systematically presents the visually perceived colors of the four anthracene derivatives, predicted based on complementary color theory. The calculations reveal that pristine anthracene (en-1, λ_max_ = 438 nm) absorbs blue light and appears yellow; the ethyl-substituted en-2 (441 nm) exhibits a negligible color shift, remaining yellow, indicating that the weak electronic effect and steric bulk of alkyl groups fail to significantly perturb its π-conjugated framework. In stark contrast, the vinyl-substituted en-3 (496 nm) is predicted to appear orange. This pronounced bathochromic shift is fully consistent with its strong intramolecular charge transfer (ICT) character, as evidenced by its narrow HOMO-LUMO gap (3.13 eV) and distinctive electron density difference (EDD) map. The vinyl group, acting as a potent electron-donating and conjugation-extending substituent, effectively lowers the excitation energy. The phenyl-substituted en-4 (464 nm) is predicted to appear red, with a redshift magnitude intermediate between en-2 and en-3, aligning with its partially hindered conjugation: while the phenyl rings extend the π-system, their torsional angle reduces electronic delocalization efficiency, resulting in an intermediate energy gap (3.42 eV) and absorption wavelength. Collectively, the color gradient in Figure 7 (yellow → yellow → orange → red) is not merely a spectral observation but a direct macroscopic manifestation of the underlying molecular electronic structure—including conjugation extent, substituent electronic effects, and spatial configuration—thereby validating the internal consistency and physical soundness of our “computation → spectra → color” closed-loop prediction model [28].

### 2.5. Electron Density Difference

**Figure 8:**
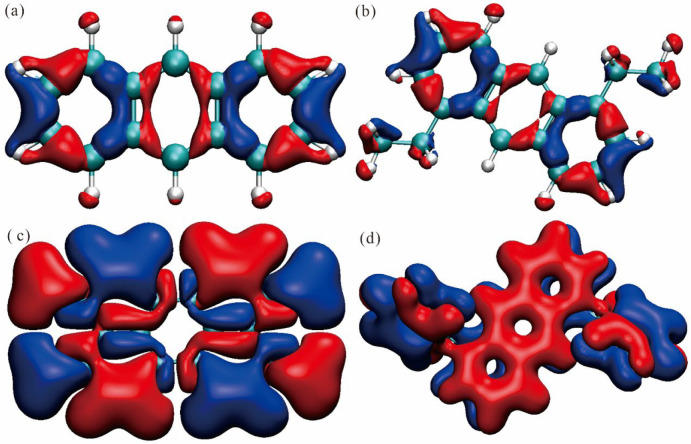
Isosurface maps of the electron density difference (Δρ) for the four compounds: (**a**) en-1; (**b**) en-2; (**c**) en-3; (**d**) en-4. As shown in the figure, the negative phase is depicted in blue, while the positive phase is shown in red.

Figure 8 presents the electron density difference (EDD) isosurface maps for anthracene and its three derivatives, rendered at an isovalue of ±0.01 e/Å^3^. In these visualizations, red regions signify an increase in electron density (ρ(S_1_) > ρ(S_0_)), while blue regions denote a decrease (ρ(S_1_) < ρ(S_0_)). We can clearly observe that the introduction of substituents acts like casting stones into the molecule’s electronic sea, generating complex and distinct ripple patterns. For pristine anthracene (en-1), the charge flow exhibits a highly symmetric pattern, with electrons migrating from both ends of the molecule toward the central region. Upon introducing ethyl groups (en-2), this symmetry is disrupted, and the charge redistribution becomes more localized; electrons flow in and out of specific areas near the substituents, revealing the subtle perturbation and inductive effect exerted by alkyl groups on the electron cloud. In stark contrast, the introduction of vinyl groups (en-3) induces a dramatic transformation. Their strong conjugating ability facilitates long-range, substantial migration of electron density between the molecular skeleton and the substituents, effectively “pulling” electrons from one side of the molecule and “depositing” them conspicuously on the other, thereby establishing a powerful, through-molecule charge separation. In comparison, phenyl substitution (en-4), while also extending the conjugated system, exhibits a more moderate and constrained charge transfer. The potential torsional angle between the phenyl and anthracene rings appears to hinder perfect planar conjugation, causing the electron flow pathways to seem partially “blocked” or “dispersed”.

**Figure 9:**
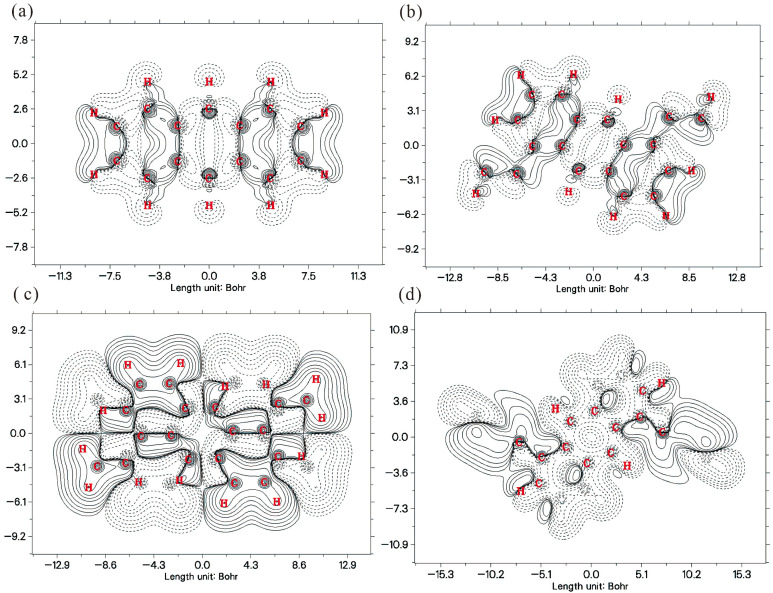
Planar contour maps of the electron density difference (Δρ) for the four. molecules: (**a**) en-1; (**b**) en-2; (**c**) en-3; (**d**) en-4. Atomic numbers are marked in red. For the lines, solid corresponds to an increase in electron density, and dashed to a decrease.

To further deepen this analysis, Figure 9 displays the corresponding planar contour maps of the electron density difference. In these plots, solid lines represent contours of increasing electron density (positive contours), while dashed lines indicate decreasing density (negative contours). Compared to the isosurface plots, the planar maps offer a clearer view of the spatial distribution details of the electron density changes. Figure 9 provides a precise two-dimensional slice, effectively “dissecting” the complex three-dimensional charge flows depicted in Figure 8 to reveal their underlying fine structure. On the plane of the parent anthracene (en-1), the pattern formed by solid and dashed lines is highly symmetric, clearly delineating the path of electrons migrating from the periphery toward the central ring. For the divinyl derivative (en-3), the planar map is particularly striking, with dense, alternating bands of solid and dashed lines running along the molecular long axis, forming a distinct “push-pull” architecture. This vividly confirms its most pronounced intramolecular charge transfer (ICT) character, which is the fundamental cause of its largest bathochromic shift in the absorption spectrum. The contours for the diethyl derivative (en-2) are comparatively sparse and localized, indicating a weaker degree of charge transfer. The contour map for the diphenyl derivative (en-4) reveals an intermediate state between the parent anthracene and the divinyl derivative; although the spatial extent of its charge redistribution is broad, its intensity is less than that of en-3, consistent with its reduced conjugation efficiency due to steric hindrance. Together, these two figures construct a comprehensive thermodynamic picture, spanning from the macro to the micro and from three-dimensional to two-dimensional perspectives, profoundly illustrating how different substituents ultimately modulate a molecule’s optical properties by altering the distribution and fluidity of its electron cloud.

## 3. Methods

All molecular models were built with Gaussian 16 [29] and GaussView 6.0 [30]. Ground-State Geometry and Properties. Molecular geometries were fully optimized and vibrational frequencies computed (to confirm absence of imaginary frequencies) using the B3LYP functional [31] with the def2TZVP basis set [32,33]. A frequency scaling factor of 0.96 was applied. Single-point energies, thermodynamic corrections, frontier molecular orbital (FMO) [34,35,36] energies (HOMO/LUMO), and the HOMO-LUMO gap were extracted from these calculations. Molecular electrostatic potential (ESP) is a central concept in wavefunction analysis and provides a quantitative description of the electrostatic interaction capability of a molecule [37,38,39]. Visualization and post-processing relied on Multiwfn 3.8 [40,41] and VMD 1.9.3 [42].

Excited-state calculations were carried out with the CAM-B3LYP functional [43] and the same def2TZVP basis set. The Gaussian input included the keywords IOp(9/40 = 4) TD (nstates = 20). After each TD-DFT run, the resulting out file was loaded into Multiwfn and the following sequence was executed: 11 → spectrum plotting, 3 → UV–Vis, 25 → color prediction from spectrum. The procedure delivered the final color predictions for indigo and its derivatives. Geometry optimizations were conducted in the gas phase, while TD-DFT calculations for excited states utilized the IEFPCM continuum model with water as the solvent to account for solvation effects relevant to practical applications.

Electron density difference (EDD) analysis [44,45]. To analyze charge redistribution upon excitation, electron density difference maps (Δρ = ρ(S_1_) − ρ(S_0_)) were computed. The ground-state (S_0_) density used the B3LYP/def2TZVP level, while the excited-state (S_1_) density used CAM-B3LYP/def2TZVP. EDD was visualized as isosurface maps and planar contour plots on the molecular XY plane (Z = 0) using a 200 × 200 grid, generated collaboratively by Multiwfn 3.8 [40,41] and VMD 1.9.3 [42].

## 4. Conclusions

This study complements and extends previous work on anthracene derivatives by providing the first systematic comparison of alkyl, vinyl, and phenyl substituent effects using consistent computational methodology. While earlier studies focused primarily on amino and isocyano substituents [46], our work establishes quantitative relationships for a broader range of functional groups.

Multiscale quantum-chemical modeling was employed to systematically elucidate the substituent-driven modulation of electronic structure and optical properties in anthracene-based dyes, successfully establishing a high-fidelity “computation → spectra → color” closed-loop prediction model. Our analysis reveals that ethyl, vinyl, and phenyl substituents cooperatively tune the HOMO–LUMO gap through combined electronic and steric effects: the vinyl group, acting as a potent electron donor with strong conjugation-extending capability, significantly narrows the gap to 3.1300 eV, inducing the largest bathochromic shift (496 nm) and predicting an orange hue; the phenyl group, while extending the π-system, introduces steric hindrance that disrupts planarity, thereby enlarging the gap to 3.4201 eV, resulting in a hypsochromic shift (464 nm) and a predicted red appearance; the ethyl group, exhibiting weak electronic influence and steric shielding, minimally perturbs the gap (3.5576 eV) and retains a yellow color. Quantitative electrostatic potential (ESP) analysis further demonstrates that vinyl substitution yields the highest surface potential variance (36.28 (kcal·mol^−1^)^2^) and maintains a relatively high polar surface area (35.16%), confirming its strong charge delocalization character. In contrast, phenyl and ethyl substitutions substantially increase the nonpolar surface area (>72%), reflecting their shielding effect on the core π-system. Electron density difference (EDD) maps vividly illustrate the pronounced push–pull intramolecular charge transfer facilitated by the vinyl group, which is fully consistent with the TD-DFT-predicted high molar extinction coefficient and long-wavelength absorption. We unify FMO, ESP, and EDD analyses into a single predictive platform for rational anthracene dye design. It provides a robust theoretical foundation and methodological paradigm for the precision engineering of high-performance OLED emitters and dye-sensitized solar cell sensitizers.

## Figures and Tables

**Table 1:** Analysis data on molecular surface electrostatic potential of anthracene and its derivatives.

Parameter	en-1	en-2	en-3	en-4
Overall Average/kcal·mol^−1^	0.41745	0.64212	0.64160	−0.00172
Positive Average/kcal mol^−1^	8.49458	6.04357	8.78666	7.24483
Negative Average/kcal·mol^−1^	–7.91073	–8.70221	–7.52192	–7.24501
Overall Variance/(kcal·mol^−1^)^2^	31.20947	28.70497	36.28215	27.18365
Positive Variance/(kcal·mol^−1^)^2^	17.60668	10.93180	23.84217	14.07184
Negative Variance/(kcal·mol^−1^)^2^	13.60279	17.77318	12.43997	13.11180
Charge Balance (ν)	0.24589	0.23580	0.22531	0.24969
Internal Charge Separation/kcal·mol^−1^	8.20431	6.85638	8.16419	7.24492
Molecular Polarity Index/kcal·mol^−1^	8.20712	7.01744	8.15501	7.24492
Nonpolar surface area (|ESP| ≤ 10 kcal/mol)	59.02%	74.23%	64.84%	72.63%
Polar surface area (|ESP| > 10 kcal/mol)	40.98%	25.77%	35.16%	27.37%

## Data Availability

The data presented in this study are available upon request from the corresponding authors.

## References

[1] Lee Y., Hwang K.M., Lee S., Park B.B., Kim T., Han W.-S. (2023). Substituent effect on anthracene-benzophenone triad system: Photophysical properties and application to OLEDs with “hot-exciton” characteristics. Dye. Pigment..

[2] Kastrati A., Oswald F., Scalabre A., Fromm K.M. (2023). Photophysical Properties of Anthracene Derivatives. Photochem.

[3] Wang R., Zhu Y., Hu D., Hu J., Chen S.-W., Lin J., Xing L., Huo Y., Ji S. (2025). Phosphorus-oxygen modified anthracene-based emitters for high-efficiency deep-blue OLEDs approaching the BT.2020 blue standard. Chem. Eng. J..

[4] Liu B., Yu Z.W., He D., Li M.D., Xie W.F., Tong Q.X. (2022). Productive harvesting of triplet excitons in anthracene-based emitters toward high-performance deep-blue nondoped organic light-emitting diodes. Mater. Today Chem..

[5] San L.K., Balser S., Reeves B.J., Clikeman T.T., Chen Y.-S., Strauss S.H., Boltalina O.V. (2025). Deep-blue emitting 9,10-bis(perfluorobenzyl)anthracene. Beilstein J. Org. Chem..

[6] Qin G.-Y., Sun X.-Q., Lin P.-P., Wei X., Guo J.-F., Cui W.-B., Fan J.-X., Li H., Zou L.-Y., Ren A.-M. (2023). The effect of heteroatoms at end groups of anthracene derivatives on the photoelectric properties and crystal/film morphology: A theoretical perspective. J. Mater. Chem. C.

[7] Al-Shamiri H.A.S. (2023). Design and construction of tunable solid-state dye laser pumped by flashlamp. Int. J. Mod. Phys. B.

[8] Wang Z., Zhang Y., Song J., Li M., Yang Y., Gu W., Xu X., Xu H., Wang S. (2019). A highly specific and sensitive turn-on fluorescence probe for hypochlorite detection based on anthracene fluorophore and its bioimaging applications. Dye. Pigment..

[9] Sahar H., Asif M., Ahsan A., Aetizaz M., Ayub K. (2024). Band gap engineering of 9,10-(bis-4-phenylazenyl)anthracene for application in dye sensitized solar cells. Sol. Energy.

[10] Zainuri D.A., Abdullah M., Bakar M.A.A., Arshad S., Razak I.A. (2023). An experimental and DFT investigations of fused ring anthracenyl chalcones with effective modification in acceptor moiety: Nonlinear optical and optical limiting response. Int. J. Quantum Chem..

[11] Hütter M., Kröger M. (2006). Phoretic forces on convex particles from kinetic theory and nonequilibrium thermodynamics. J. Chem. Phys..

[12] De Melo J.S.S., Rondão R., Burrows H.D., Melo M.J., Navaratnam S., Edge R., Voss G. (2006). Spectral and Photophysical Studies of Substituted Indigo Derivatives in Their Keto Forms. ChemPhysChem.

[13] Ikeda K., Yoo D., Nishikawa R., Kawamoto T., Mori T. (2021). Charge injected proton transfer in indigo derivatives. Phys. Chem. Chem. Phys..

[14] Costa R.F., Oliveira M.S., Aguiar A.S.N., Custodio J.M.F., Di Mascio P., Sabino J.R., Verde G.V., Souza J.C., Santin L.G., Camargo A.J. (2021). Synthesis and Structural Studies of Two New Anthracene Derivatives. Crystals.

[15] Dai Y., Liu H., Geng T., Duan R., Li X., Liu Y., Liu W., He B.-G., Sui L., Wang K. (2023). Piezochromic fluorescence of anthracene derivative crystals with different stacking patterns designed around excimers. J. Mater. Chem. C.

[16] Lu Z., Li C., Li S., Yu Q., Zhang J. (2025). DFT Study on Fused N-Heteroaromatic Frameworks: Stability, Aromaticity, and Energetic Insights from Five-Membered Fused Six-Membered N-Heteroaromatic Skeletons. Molecules.

[17] Wen Y., Yu Y., Gu H., Kang Y., Zhao G., Li Y., Huang Q. (2025). First-Principles Study of Rh Segregation in the Au–Rh(111) Alloy with Adsorbed NO, CO, or O_2_. Molecules.

[18] Piękoś P., Lipkowski P., Dehaen W., Wieczorek R., Filarowski A. (2025). Theoretical Insights into the Impact of Pyrrole and Imidazole Substituents on the BODIPY Chromophore. Molecules.

[19] De Paul Zoua V., Atangana A.F., Asi A.Q., Pagoré I.F., Tatsimo S.J.N., Ntieche R.A. (2024). Structural, electronic, and NLO properties of two acridone alkaloîds: DFT and TD-DFT studies. J. Mol. Model..

[20] Kushwaha P.K., Srivastava S.K. (2024). Tuning optoelectronic properties of indandione-based D-A materials by malononitrile group acceptors: A DFT and TD-DFT approach. J. Mol. Model..

[21] Li X., Lu H., He D., Luo C., Huang J. (2013). Synthesis, Fluorescence Properties and Theoretical Calculations of Novel Stilbene Derivatives Based on 1,3,4-Oxadiazole Bearing Anthracene Core. J. Fluoresc..

[22] Luo Y., Tao S., Wu Y., Feng W., Jiang W., Xia Y., Xiao W., Li Y., Liu Z., Ou Y.-P. (2024). Donor-acceptor type dithienylethenes derivatives based on anthraquinone and anthracene moieties: Synthesis, photochromic properties and theoretical calculations. Dye. Pigment..

[23] Hleli E., Mbarek M., Gouid Z., Ulbricsht C., Romdhane S., Ben Said R., Guesmi M., Egbe D.A.M., Bouchriha H. (2020). DFT study of optical and electronic properties of anthracene containing PPE-PPVs. J. Phys. Chem. Solids.

[24] Alrub S.A., Shah Y., Umar M., Mansha A., Ali A.I., Hussein R.K. (2025). Theoretical investigations of the auxochromic effect on novel thermally activated delayed fluorescence (TADF) anthracene derivatives. BMC Chem..

[25] Cysewski P., Jeliński T., Przybyłek M., Shyichuk A. (2012). Color prediction from first principle quantum chemistry computations: A case of alizarin dissolved in methanol. New J. Chem..

[26] Jeon B.K., Jang S.H., Kim S.H., Lee H., Choi I., Lee B.-S., Kang K., Choi J. (2025). Development of electron-flow-strengthened azo dyes with a wide color reproduction range for application in the color conversion layers of microdisplays. Dye. Pigment..

[27] Trussell H.J. (1993). DSP solutions run the gamut for color systems. IEEE Signal Process. Mag..

[28] Aroca-Trujillo J.L., Perez-Ruiz A. (2025). Colombian coffee tree leaves multispectral images dataset. Data Brief..

[29] Frisch M.J., Trucks G.W., Schlegel H.B., Scuseria G.E., Robb M.A., Cheeseman J.R., Scalmani G., Barone V., Petersson G.A., Nakatsuji H. (2016). Gaussian 16.

[30] Dennington R., Keith T.A., Millam J.M. (2016). GaussView.

[31] Stephens P.J., Devlin F.J., Chabalowski C.F., Frisch M.J. (1994). Ab Initio Calculation of Vibrational Absorption and Circular Dichroism Spectra Using Density Functional Force Fields. J. Phys. Chem..

[32] Weigend F., Ahlrichs R. (2005). Balanced basis sets of split valence, triple zeta valence and quadruple zeta valence quality for H to Rn: Design and assessment of accuracy. Phys. Chem. Chem. Phys..

[33] Gulde R., Pollak P., Weigend F. (2012). Error-Balanced Segmented Contracted Basis Sets of Double-ζ to Quadruple-ζ Valence Quality for the Lanthanides. J. Chem. Theory Comput..

[34] Zhang Z.-F., Su M.-D. (2025). Insights into the Factors Controlling the Origin of Activation Barriers in the [2 + 2] Cycloaddition Reactions of Heavy Imine-like Molecules Featuring a Ge=Group 15 Double Bond with Heterocumulenes. Molecules.

[35] Suzuki K., Inoue K., Namiguchi R., Morita S., Hayakawa S., Yokota M., Sakai K., Matsumoto K., Aoki S. (2025). Identification of Novel Compounds That Bind to the HGF β-Chain In Silico, Verification by Molecular Mechanics and Quantum Mechanics, and Validation of Their HGF Inhibitory Activity In Vitro. Molecules.

[36] Paciotti R., Re N., Storchi L. (2024). Combining the Fragment Molecular Orbital and GRID Approaches for the Prediction of Ligand–Metalloenzyme Binding Affinity: The Case Study of hCA II Inhibitors. Molecules.

[37] Manzetti S., Lu T. (2013). The geometry and electronic structure of Aristolochic acid: Possible implications for a frozen resonance. J. Phys. Org. Chem..

[38] Lu T., Chen F. (2012). Quantitative analysis of molecular surface based on improved Marching Tetrahedra algorithm. J. Mol. Graph. Model..

[39] Zhang J., Lu T. (2021). Efficient evaluation of electrostatic potential with computerized optimized code. Phys. Chem. Chem. Phys..

[40] Lu T., Chen F. (2012). Multiwfn: A multifunctional wavefunction analyzer. J. Comput. Chem..

[41] Lu T. (2024). A comprehensive electron wavefunction analysis toolbox for chemists, Multiwfn. J. Chem. Phys..

[42] Humphrey W., Dalke A., Schulten K. (1996). VMD: Visual molecular dynamics. J. Mol. Graph..

[43] Li M., Reimers J.R., Ford M.J., Kobayashi R., Amos R.D. (2021). Accurate prediction of the properties of materials using the CAM-B3LYP density functional. J. Comput. Chem..

[44] Wahl A.C. (1966). Molecular Orbital Densities: Pictorial Studies. Science.

[45] Schwarz W.H.E., Mensching L., Valtaznos P., Von Niessen W. (1986). A chemically useful definition of electron difference densities. Int. J. Quantum Chem..

[46] Nagy M., Fiser B., Szőri M., Vanyorek L., Viskolcz B. (2022). Optical Study of Solvatochromic Isocyanoaminoanthracene Dyes and 1,5-Diaminoanthracene. Int. J. Mol. Sci..

